# A Selection Operator for Summary Association Statistics Reveals Allelic Heterogeneity of Complex Traits

**DOI:** 10.1016/j.ajhg.2017.09.027

**Published:** 2017-12-05

**Authors:** Zheng Ning, Youngjo Lee, Peter K. Joshi, James F. Wilson, Yudi Pawitan, Xia Shen

**Affiliations:** 1 Department of Medical Epidemiology and Biostatistics, Karolinska Institutet, Nobels väg 12A, SE-171 77 Stockholm, Sweden; 2 Department of Statistics, Seoul National University, Seoul 151747, South Korea; 3 Center for Population Health Sciences, Usher Institute of Population Health Sciences and Informatics, Old Medical School, University of Edinburgh, Teviot Place, Edinburgh EH8 9AG, Scotland, United Kingdom; 4 Medical Research Council Human Genetics Unit, MRC Institute of Genetics and Molecular Medicine, University of Edinburgh, Western General Hospital, Crew Road, Edinburgh EH4 2XU, Scotland, United Kingdom

**Keywords:** LASSO, summary association statistics, genome-wide association study, complex traits, fine-mapping, prediction, shrinkage estimator, allelic heterogeneity, missing heritability, human height

## Abstract

In recent years, as a secondary analysis in genome-wide association studies (GWASs), conditional and joint multiple-SNP analysis (GCTA-COJO) has been successful in allowing the discovery of additional association signals within detected loci. This suggests that many loci mapped in GWASs harbor more than a single causal variant. In order to interpret the underlying mechanism regulating a complex trait of interest in each discovered locus, researchers must assess the magnitude of allelic heterogeneity within the locus. We developed a penalized selection operator for jointly analyzing multiple variants (SOJO) within each mapped locus on the basis of LASSO (least absolute shrinkage and selection operator) regression derived from summary association statistics. We found that, compared to stepwise conditional multiple-SNP analysis, SOJO provided better sensitivity and specificity in predicting the number of alleles associated with complex traits in each locus. SOJO suggested causal variants potentially missed by GCTA-COJO. Compared to using top variants from genome-wide significant loci in GWAS, using SOJO increased the proportion of variance prediction for height by 65% without additional discovery samples or additional loci in the genome. Our empirical results indicate that human height is not only a highly polygenic trait, but also has high allelic heterogeneity within its established hundreds of loci.

## Introduction

Genome-wide association studies (GWASs) have successfully identified many genetic variants that regulate complex traits. However, the associations between a complex trait and genetic variants, such as single-nucleotide polymorphisms (SNPs), are usually very small relative to noise. Thus, GWASs often require large sample sizes to achieve sufficient power, and substantial efforts have been spent on the development of statistical methods to boost GWAS discovery power.

Given the legal restrictions on public sharing of individual-level data, it is rarely feasible to pool individual-level data from a number of different cohorts. In spite of this, GWAS summary-level data, in the form of association statistics, are mostly meta-analyzed and reported.^1^ Hence, the recent focus in methodology has been on meta-analysis techniques that use summary-level data based on established results to extract further knowledge. Based on association statistics, a few state-of-the-art methods, such as summary-level Mendelian randomization (SMR) analysis for candidate-gene-target prediction;^2^ LD score regression (LDSC) for polygenicity detection, heritability, and genetic correlation estimation;^3^ and conditional and joint multiple-SNP analysis (GCTA-COJO) for detection of independent associations within quantitative trait loci (QTL) discovered in GWAS,^4^ have been developed.

In GWASs, if the single most statistically significant variant at a locus is reported, we only capture all the genetic variance—i.e., there is no missing heritability at the locus—when two assumptions hold: (1) there is only one underlying causal variant at the locus, and (2) the causal variant is in complete linkage disequilibrium (LD) with the top variant. However, these two assumptions can both be questioned: (1) there might be multiple causal variants or alleles at the locus so that a single variant cannot account for all the genetic variance at the locus. The phenomenon wherein multiple causal variants or alleles for a particular trait are located at the same locus is known as allelic heterogeneity (AH), whose presence in various complex diseases is reported in a recent study.^5^ (2) Even if there is only one underlying causal variant at the locus, a single top variant cannot capture all the genetic variance if the LD between the top variant and the causal variant is incomplete. To identify secondary association signals, many GWAS meta-analyses have used conditional analysis such as GCTA-COJO. GCTA-COJO performs a secondary association analysis conditioned on discovered top variants; such conditional analysis is conducted with GWAS meta-analysis summary statistics rather than individual-level data of the full sample. In recent analyses conducted by global consortia such as GIANT and DIAGRAM, GCTA-COJO was successful in detecting multiple associations in LD at the same loci.^6^, ^7^, ^8^, ^9^

However, the forward stepwise selection procedure, such as that implemented in GCTA-COJO, is known to be overly “greedy”; it is prone to eliminating useful predictors that happen to be correlated with selected predictors.^10^ This indicates that GCTA-COJO might miss some informative variants as a result of their LD with detected variants. More variants can be discovered when the discovery p value threshold is less stringent in GCTA-COJO. But as a fixed-effect model-selection strategy, there is a risk of overfitting for GCTA-COJO, especially when too many predictors are included in the model as p value threshold is increased.

There is theoretical and empirical evidence that simultaneous modeling of multiple predictors with penalization provides a better variable selection procedure than the forward stepwise selection.^11^ In this framework, the least absolute shrinkage and selection operator (LASSO)^12^ was introduced and applied to variable selection problems in various disciplines.^13^, ^14^ Instead of only considering the square loss function $\left(1/2\right){\left\|y-X\hat{\beta }\right\|}_{2}^{2}$, LASSO takes the $\ell_{1}$-norm regularization ${\left\|\hat{\beta }\right\|}_{1}$ into account and solves

$$\underset{\hat{\beta }\in R^{p}}{\text{min}}\;\frac{1}{2}{\|y-X\hat{\beta }\|}_{2}^{2}+\lambda {\|\hat{\beta }\|}_{1},$$

where the tuning parameter λ≥0. Intuitively, the $\ell_{1}$ term is a penalization: the larger λ is, the larger the penalty imposed on the coefficients. This makes LASSO allow large coefficients only when they lead to a substantially better fit. LASSO leads to better interpretability and prediction accuracy.^12^ Because of $\ell_{1}$ regularization, LASSO has the ability to perform variable selection and get parsimonious results. Besides, as a shrinkage method, LASSO alleviates overfitting problems by performing a more reasonable bias-variance trade-off, which allows LASSO to include more informative predictors in the model without serious overfitting. The LARS algorithm^10^ and regularization path algorithm^15^ provide computationally fast ways for solving the LASSO. These benefits make LASSO potentially highly useful in genetics research. In many recent papers, LASSO was used for selecting variants^16^ and building prediction models.^17^

The aim of this study is to develop, implement, and validate LASSO by using GWAS summary statistics (SOJO) for genomic loci discovered in standard GWASs. First, we show that using summary-level data for LASSO achieves results that are approximately equivalent to those obtained when LASSO is based on individual-level data. We then provide simulation studies to show how SOJO can outperform GCTA-COJO in finding additional association signals in loci with different LD structures. We applied SOJO on GWAS summary-level data of three anthropometric traits—height, body mass index (BMI), and waist-to-hip ratio after adjustment for body mass index (WHRadjBMI) reported by the GIANT consortium—and validated the out-of-sample predictive performance in the large national cohort UK Biobank (UKB). By implementing SOJO, we have added additional association information to the results of standard GWASs and GCTA-COJO analyses, improved out-of-sample predictive heritability, and revealed different levels of allelic heterogeneity for different traits. The SOJO analysis is implemented in our free and open-source R package.

## Material and Methods

### LASSO Regularization Path Based on GWAS Summary Statistics

In this section, we describe how to achieve LASSO estimates by using summary-level statistics from a GWAS meta-analysis and a reference sample. Assume a quantitative trait *y* is potentially affected by a group of genetic variants $X_{1},\ldots ,X_{p}$ and a multi-variant linear model

(Equation 1) y=Xβ+e,

where $X=\left(X_{1},\ldots ,X_{p}\right)$. If we have *n* individuals, then $y=\left\{y_{i}\right\}$ is the n×1 phenotype vector, and $X=\left\{x_{ij}\right\}$ is the n×p genotype matrix. To get an estimate of regression coefficients $\hat{\beta }=\left({\hat{\beta }}_{1},\ldots ,{\hat{\beta }}_{p}\right)$, we look at the square loss function $\left(1/2\right){\left\|y-X\hat{\beta }\right\|}_{2}^{2}$ and the $\ell_{1}$-norm regularization ${\left\|\hat{\beta }\right\|}_{1}$, which leads to the LASSO optimization problem

(Equation 2) $$\underset{\hat{\beta }\in R^{p}}{\text{min}}\;\frac{1}{2}{\|y-X\hat{\beta }\|}_{2}^{2}+\lambda {\|\hat{\beta }\|}_{1},$$

where the tuning parameter λ≥0.

The regularization path^10^ can be used to compute LASSO estimates in Equation 2 as a function of λ, denoted by $\hat{\beta }\left(\lambda \right)$, for all λ∈[0,∞]. Interestingly, when the sample size is large, the regularization-path algorithm only depends on (1) the covariance structure between variants and the trait, and (2) the LD structure between variants. Therefore, we can approximate LASSO estimates by using summary-level statistics from a GWAS meta-analysis and a reference sample.

The first step is to get the covariance structure between variants and the trait. To simplify the formulae, we center the data so that $\bar{y}=0$ and ${\bar{X}}_{j}=0$, where j=1,2,…,n, and the intercept does not need to be included. Because the centering does not affect the estimates of slope in summary-level statistics, we can take the GWAS results in meta-analysis as they are from centered data. Then in the GWAS, each variant is fitted according to a univariate regression model:

(Equation 3) $$y=X_{j}b_{j}+e.$$

Based on Equation 3, the marginal effect of variant *j* is

(Equation 4) $${\hat{b}}_{j}={\left(X_{j}^{T}X_{j}\right)}^{-1}X_{j}^{T}y\approx \frac{\text{Cov}\left(X_{j},y\right)}{\text{Var}\left(X_{j}\right)},$$

and its variance is

(Equation 5) $$\sigma_{{\hat{b}}_{j}}^{2}=\sigma_{r}^{2}{\left(X_{j}^{T}X_{j}\right)}^{-1}\approx \frac{\sigma^{2}}{n\text{Var}\left(X_{j}\right)},$$

where $\sigma_{r}^{2}$ is the residual variance in univariate regression (Equation 3) and $\sigma^{2}$ is the phenotypic variance. Because the effect of a single variant is usually small, we can approximate $\sigma_{r}^{2}$ by $\sigma^{2}$. From Equation 4 and Equation 5, we have

(Equation 6) $$\hat{\text{Cov}\left(X_{j},y\right)}=\frac{{\hat{b}}_{j}\sigma^{2}}{\sigma_{{\hat{b}}_{j}}^{2}n},$$

where all terms on the right side except $\sigma^{2}$ are reported in the GWAS meta-analysis results. For $\sigma^{2}$, because all $\hat{\beta }$s and λ in the algorithm are proportional to $\sigma^{2}$, it is fine to assume $\sigma^{2}=1$ if only the variable selection or $R^{2}$ explained by polygenic scores is concerned. If exact estimates of coefficients are needed, $\sigma^{2}$ can be estimated by the phenotypic variance in the reference sample mentioned below.

The LD structure between variants can be approximated by a representative reference sample where individual-level genotype data are available.^4^ A proper reference sample can be a cohort included in the meta-analysis study. Let $W=\left\{w_{ij}\right\}$ represent the $n_{W}\times p$ genotype matrix of the reference sample. Then

(Equation 7) $$\hat{\text{Var}\left(X\right)}=\text{Var}\left(W\right).$$

To simplify symbols, we define $C_{p\times 1}=\hat{\text{Cov}\left(X,y\right)}$ and $B_{p\times p}=\hat{\text{Var}\left(X\right)}$. Considering different allele frequency between variants, we suggest using C and B with standardized X. Let $D_{\text{W}}$ denote the diagonal matrix of Var(W). Standardized X leads to

(Equation 8) $$C=D_{\text{W}}^{-1/2}\hat{\text{Cov}\left(X,y\right)}$$

(Equation 9) $$B=D_{\text{W}}^{-1/2}\hat{\text{Var}\left(X\right)}D_{\text{W}}^{-1/2}.$$

Let *k* be the step counter, $\lambda_{k}$ be the tuning parameter at the current step, $s_{j}$ denote the sign of ${\hat{\beta }}_{j}$, and $A=\left\{j:s_{j}\neq 0\right\}$ be the active set. Starting with $k=0,\lambda_{0}=\infty$, and A=ϕ. The LASSO regularization path algorithm can be implemented as follows;

1. Get the next hitting time

(Equation 10) $$\lambda_{k+1}^{\text{hit}}=\underset{j\notin A,s_{j}\in \left\{-1,1\right\}}{{\text{max}}^{+}}\frac{X_{j}^{T}y-X_{j}^{T}X_{A}{\left(X_{A}^{T}X_{A}\right)}^{-1}X_{A}^{T}y}{n\left(s_{j}-X_{j}^{T}X_{A}{\left(X_{A}^{T}X_{A}\right)}^{-1}s_{A}\right)}$$

(Equation 11) $$\approx \underset{j\notin A,s_{j}\in \left\{-1,1\right\}}{{\text{max}}^{+}}\frac{C_{j}-B_{jA}B_{A}^{-1}C_{A}}{\left(s_{j}-B_{jA}B_{A}^{-1}s_{A}\right)},$$

where ${\text{max}}^{+}$ means the maximum argument that is smaller than $\lambda_{k}$. Denote the index of the hitting variable as $h_{k}$ and its sign as $s_{h_{k}}$. Specifically,

(Equation 12) $$\lambda_{1}^{\text{hit}}=\underset{j}{\text{max}}\frac{\left|X_{j}^{T}y\right|}{n}\approx \underset{j}{\text{max}}|C_{j}|.$$

2. Get the next crossing time

(Equation 13) $$\lambda_{k+1}^{\text{cross}}=\underset{j\in A}{{\text{max}}^{+}}\frac{{\left[{\left(X_{A}^{T}X_{A}\right)}^{-1}X_{A}^{T}y\right]}_{j}}{n{\left[{\left(X_{A}^{T}X_{A}\right)}^{-1}s_{A}\right]}_{j}}$$

(Equation 14) $$\approx \underset{j\in A}{{\text{max}}^{+}}\frac{{\left[B_{A}^{-1}C_{A}\right]}_{j}}{{\left[B_{A}^{-1}s_{A}\right]}_{j}},$$

where ${\text{max}}^{+}$ means the maximum argument that is smaller than $\lambda_{k}$. Denote the index of the crossing variable as $c_{k}$ and its sign as $s_{c_{k}}$. Specifically, $\lambda_{1}^{\text{cross}}=0$.

3. Let

$$\lambda_{k+1}=\text{max}\left\{\lambda_{k+1}^{\text{hit}},\lambda_{k+1}^{\text{cross}}\right\}.$$

If $\lambda_{k+1}^{\text{hit}}\geq \lambda_{k+1}^{\text{cross}}$, then add the index of the hitting variable $h_{k}$ to A and its sign $s_{h_{k}}$ to $s_{A}$. If $\lambda_{k+1}^{\text{hit}}<\lambda_{k+1}^{\text{cross}}$, then remove the index of the crossing variable $c_{k}$ from A and its sign $s_{c_{k}}$ from $s_{A}$.

4. Get the LASSO estimate at $\lambda_{k+1}$ from

(Equation 15) $${\hat{\beta }}_{A}\left(\lambda_{k+1}\right)={\left(X_{A}^{T}X_{A}\right)}^{-1}\left(X_{A}^{T}y-\lambda_{k+1}s_{A}\right)$$

(Equation 16) $$\approx B_{A}^{-1}\left(C_{A}-\lambda_{k+1}s_{A}\right)$$

(Equation 17) $${\hat{\beta }}_{j}\left(\lambda_{k+1}\right)=0,\;\text{for all }j\notin A.$$

5. Then update *k* to k+1 and repeat steps 1–4 until $\lambda_{k+1}=0$.

If the standardized X is used, and if the coefficients under standardization with tuning parameter λ are denoted as ${\hat{\beta }}_{s}\left(\lambda \right)$, then the coefficients on the original scale

(Equation 18) $$\hat{\beta }\left(\lambda \right)=D_{\text{W}}^{-1/2}{\hat{\beta }}_{s}\left(\lambda \right),$$

for any λ∈[0,∞].

In GWAS meta-analysis results, the sample sizes for different variants are usually different because of imputation failures in the studies involved. However, $\text{Cov}\left(X_{j},y\right)$ is estimated for each variant separately in Equation 6. Therefore, the above algorithm is still valid.

### Summary Statistics of Anthropometric Traits and Individual-Level Genotype Data

The GIANT Consortium performed a GWAS meta-analysis by using the summary statistics from 79, 125, and 101 studies, consisting of 253,288, 322,154, and 210,088 individuals of European ancestry for adult height,^6^ BMI,^7^ and WHRadjBMI,^8^ respectively. Meta-analysis was performed on ∼2.6 million SNPs for all the three traits. After SNPs with MAF < 0.01 were excluded, ∼2.5 million SNPs remained. Considering the accuracy of the estimated correlation between SNPs and traits, we excluded SNPs with sample size less than 2/3 of the maximum sample size but retained ∼2.4 million, ∼2.2 million, and ∼1.7 million SNPs for height, BMI, and WHRadjBMI, respectively. We also used the individual-level genotype data of the TwinGene cohort, which is a population-based Swedish study of twins born between 1911 and 1958.^18^ Genotyping was done with the Illumina OmniExpress BeadChip. After the quality control, 644,556 SNPs and 9,617 individuals remained, including all available dizygotic twins and one twin from each available monozygotic twin pair. Another source of individual-level genotype data is the 503 European ancestry samples in 1000 Genomes Project phase 3 data.^19^

### UK Biobank Data

The UK Biobank recruited 500,000 people aged 40–69 years between 2006 and 2010 from across the country. Here, a wave 1 public release in June 2015 is used. Among individuals whose phenotypic information was available, 152,732 had been genotyped on an Affymetrix chip that included about 800,000 variants. Millions of further variants were imputed. Among the genotyped individuals, 120,286 were identified as genetically British by the UK Biobank. These individuals were taken forward for analysis in this paper. In the prediction performance analyses, height, BMI, and WHRadjBMI in UKB were adjusted for age and sex before being standardized to z-scores.

### Application of SOJO at Established Genome-wide Significant Loci

For each trait, first we took all loci with genome-wide significant SNPs reported in GIANT results. There were 423, 77, and 49 loci for height, BMI, and WHRadjBMI, respectively. For each of these loci, we set a 1 Mb window centered at the most significant variant as the genomic locus to be analyzed. We performed SOJO to select the associated variants for each locus by using the following steps:

1. We took the intersection of available variants in GIANT and TwinGene.

2. We estimated LD correlations by using individual-level genotype data in TwinGene.

3. We filtered the variants according to the LD correlation matrix. If the LD $r^{2}$ between a pair of variants was larger than 0.9, only the more significant one in GWAS meta-analysis was kept for further analysis.

4. We ran the summary-level LASSO algorithm by using summary statistics from GIANT and the filtered estimated LD correlations in step 3.

5. Along the LASSO path, the SNPs were included or removed from the model one by one as λ decreased. For each point, when the active-variant set changed, we computed the out-of-sample $R^{2}$ on the basis of the current active-variant set and coefficients.

6. We reported the variants that maximized the out-of-sample $R^{2}$ and their penalized effects.

In step 3, we removed one SNP from each pair of extremely correlated variants because (1) including both of them didn’t significantly increase the amount of information gained, and (2) including both might have generated numerical errors when the tuning parameter went to zero and the model approached the standard multiple regression.

### Adjust Model Degrees of Freedom for Comparison

For the comparison between SOJO and GCTA-COJO to be fair, the two must be under the same level of model complexity. Degrees of freedom is often used as a measurement of model complexity. When comparing two linear models, both with *p* predictors, one can say their model complexity is the same because their degrees of freedom are both equal to *p*. However, when it comes to evaluating two complex variable selection procedures, especially when comparisons or shrinkage is involved, the degrees of freedom or the complexity of the model might no longer be equal to the number of variables selected by the model.^20^ Suppose we have observations $y\in R^{n}$ where

(Equation 19) $$y=\mu +\epsilon ,\;\text{with E}\left(\epsilon \right)=0,\;\text{Cov}\left(\epsilon \right)=\sigma^{2}I.$$

For a function $f:R^{n}\to R^{n}$ producing fitted values f(y) based on y, the value of the generalized degrees of freedom (GDF)^21^ is defined as:

(Equation 20) $$\text{df}\left(f\right)=\frac{1}{\sigma^{2}}\sum_{i=1}^{n}\text{Cov}\left(f_{i}\left(y\right),y_{i}\right).$$

Estimating GDF for GCTA-COJO and SOJO with a Monte Carlo method^20^ requires individual-level data. Without GIANT individual-level data, we could not directly estimate the GDF when GIANT was the discovery sample. Instead, we saw GDF as a piecewise function of the number of selected variables, and we estimated the function by using the UKB data. First, we estimated the GDF for GCTA-COJO and SOJO locus by locus for each trait by using UKB data. We performed the estimation by using multiple p value thresholds for GCTA-COJO and different tuning parameters for LASSO. In this way, we obtained an estimate of the function mapping the number of selected variables to GDF. Then, for each variable selection result based on GIANT and TwinGene, we could estimate the GDF by using the function. According to our result, if we include *k* variables in our model, SOJO costs exactly *k* GDF, which is consistent with theoretical results, whereas GCTA-COJO usually costs more than *k* GDF.^22^ An example is given in Figure S1.

## Results

### LASSO from Summary-Level Data Approximates That from Individual-Level Data

**Figure 1:**
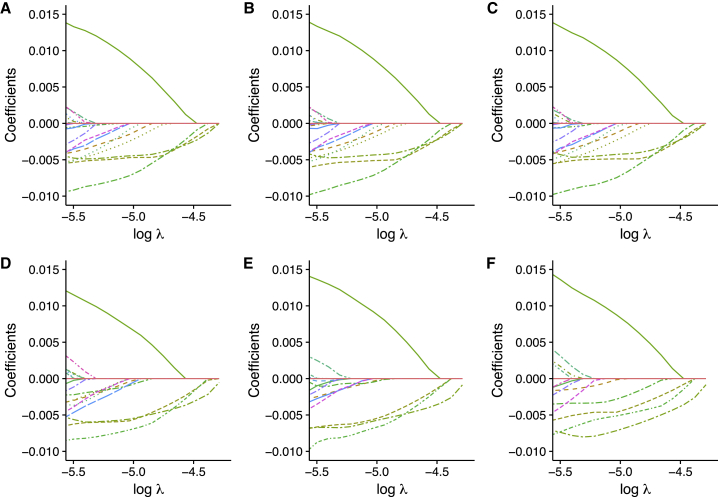
An Example Showing the Approximation of Summary-Level LASSO to Individual-Level LASSO The phenotype and genotype data are from 120,086 individuals in the UK Biobank. GWAS was performed on height. The curves represent regularization paths of Lasso coefficients. The six plots show LASSO results in six different scenarios of data: (A) LASSO based on individual level data. (B) LASSO based on GWAS summary statistics and LD correlations estimated from the whole cohort. (C) LASSO based on GWAS summary statistics and LD correlations estimated from a subcohort where n = 10,000. (D) LASSO based on unequal sample sizes, GWAS summary statistics, and LD correlations estimated from a subcohort where n = 10,000. The subcohorts in (C) and (D) are randomly sampled from the whole cohort. In (D), for each variant, a subset of individuals with random sample size between 110,000 and 120,086 was taken. Then, GWAS summary statistics were computed on the basis of the data from the unequal sample sizes. (E) The GWAS summary statistics are the same as in (D), but LD correlations are estimated from 9,617 TwinGene samples. (F) The GWAS summary statistics are the same as in (D), but LD correlations are estimated from 503 European ancestry samples in 1000 Genomes.

We can approximate the LASSO result at any tuning parameter by using (1) the covariance structure between variants and the trait and (2) the LD structure between variants. The former covariance structure can be estimated from GWAS meta-analysis summary-level data, and the LD structure can be estimated from a reference sample, such as a subcohort of the GWAS meta-analysis. Figure 1 shows the similarity of LASSO results under six different scenarios. In each plot, each line shows how the coefficient estimates vary under different tuning parameters. Theoretically, when the effects of single variants are all small and the whole cohort is taken as reference sample, the summary-level LASSO estimates are the same as those based on individual-level LASSO results (Figures 1A and 1B). A real scenario can be more complicated in two ways: (1) individual-level data are available only for a subset of the cohort, which affects the estimation of LD correlation between variants, and (2) the sample sizes are usually different for different variants because of, e.g., imputation failures in the studies involved. However, as shown in Figures 1C and 1D, when a relatively large subsample is used as the reference sample and the number of missing individuals for each variant is not substantial, the summary-level LASSO results are close to individual-level LASSO results. When the representative reference samples are outside of the discovery population, the summary-level LASSO results are still similar (Figures 1E and 1F). Our simulation shows that the out-of-sample prediction performance is also similar for these scenarios (Figure S2).

### SOJO Shows High Sensitivity in Most Types of LD Structure

**Figure 2:**
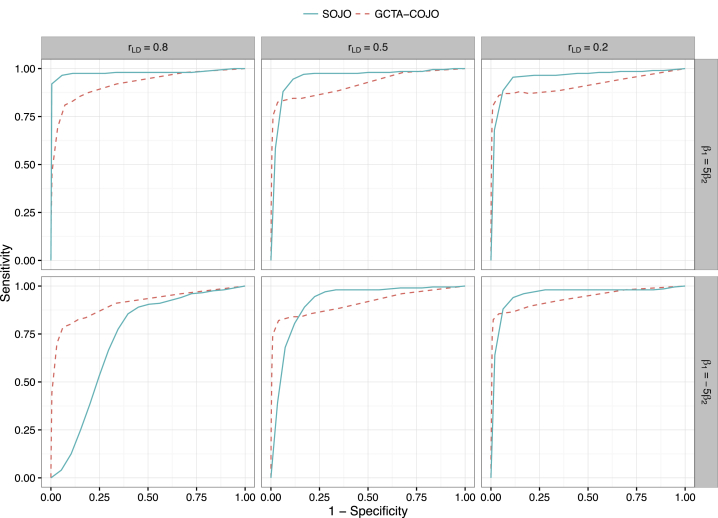
Receiver-Operating-Characteristic Curves Comparing the Performance of SOJO and GCTA-COJO for Correlated Causal-Variant Identification on Simulated Data Datasets were simulated for 100,000 individuals with 20 variants, where $\text{cor}\left(X_{i},X_{j}\right)=0.8^{\left|i-j\right|}$. The allele frequencies are all equal to 0.5. To simplify the model, assuming genotype columns are demeaned, the trait $y=\beta_{1}X_{c1}+\beta_{2}X_{c2}+e$, where $X_{c1},X_{c2}$ are causal variants and $e∼N\left(0,\sigma^{2}\right)$. In all simulations, $\beta_{1}=5$ and $\sigma^{2}=50$. $r_{\text{LD}}=\text{cor}\left(X_{c1},X_{c2}\right)$ varies from 0.8 to 0.5 and 0.2. $\beta_{2}$ is either 1 or −1. For both SOJO and GCTA-COJO, the whole sample was taken as the reference sample. For each case, 200 datasets were generated. Solid curves represent SOJO, and dashed curves represent GCTA-COJO.

We simulated a model of two correlated causal variants in order to compare SOJO and GCTA-COJO in terms of sensitivity and specificity. The area under the curve (AUC) of SOJO is larger than that of GCTA-COJO in most cases (Figure 2). The only exception is when the directions of the two genetic effects do not agree with the sign of the LD correlation between these two variants, yet the LD is strong. Namely, $\beta_{1}\times \beta_{2}\times r_{\text{LD}}<0$ and, at the same time, $r_{\text{LD}}$ is large (Figure 2, bottom-left panel). The exception is relatively unlikely in practice. This can be verified by 147 loci with more than one height-associated variant reported in the GCTA-COJO analysis of GIANT data. If we focus on the first two significant height-associated variants, the top two variants for 24 out of the 147 loci have an absolute value of correlation larger than 0.2. Among these 24 loci, only seven have a discrepancy between the sign of the LD correlation and the directions of the two genetic effects. Therefore, in this case the exception rate is 7/147.

### Analysis of Three Anthropometric Traits

In this study, we took a subcohort of *GIANT: the Swedish Twin Registry* (TwinGene, n = 9,617) as our reference sample and focused on the 644,556 chip variants in TwinGene. Using GIANT summary statistics for height, BMI, and WHRadjBMI of 253,288, 322,154, and 210,088 individuals, and data for 120,286 individuals from UKB as a validation sample, we prioritized 8,470, 1,026, and 522 jointly associated variants by implementing SOJO on 423, 77, and 49 established loci for the three traits, respectively (Table S1). On average, 20, 13, and 11 variants were selected in each locus for height, BMI, and WHRadjBMI, respectively. In each locus, we performed summary-level LASSO and reported variants and their penalized effects when out-of-sample prediction $R^{2}$ was maximized in a validation sample.

**Figure 3:**
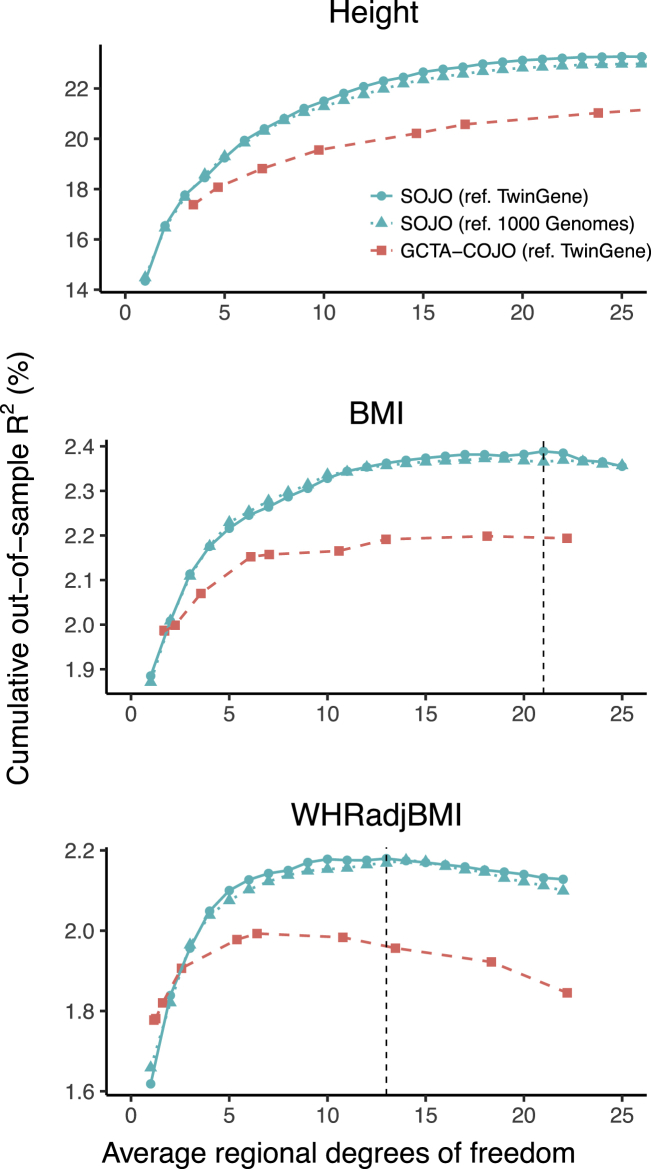
Out-of-Sample Prediction Performance Comparison of SOJO and GCTA-COJO in Terms of Height, BMI, and WHRadjBMI Solid curves represent SOJO, and dashed curves represent GCTA-COJO. The vertical dashed lines represent the average regional degrees of freedom when cumulative out-of-sample $R^{2}$ starts dropping. The x axis represents the average regional degrees of freedom, which is an estimate of the effective number of parameters in a model. For GCTA-COJO, the regional degrees of freedom are usually larger than the number of selected variants. But for SOJO, the regional degrees of freedom is equal to the number of selected variants (see Material and Methods).

To assess the performance of SOJO and GCTA-COJO, we used $R^{2}$ for cumulative out-of-sample prediction as a criterion, where GIANT and TwinGene were used for discovery and UKB for validation. For each method, we first set a universal threshold for selection: a p value cut-off for GCTA-COJO, and the number of top variants for SOJO. We then implemented the method on each trait-associated locus reported by GIANT. For each locus, given the universal threshold, a set of candidate variants and their effects were computed. Using genotypes and estimated effects of these variants, we built a polygenic score and computed the proportion of predictable variance from the regional polygenic score in UKB. We then obtained cumulative out-of-sample prediction $R^{2}$ by summing all regional proportions of explained variance (Figure 3). By setting a fixed number of selected variants for all regions, SOJO still outperforms COJO in terms of prediction performance for all three traits. SOJO achieves maximum $R^{2}$ of 23.29%, 2.39%, and 2.18% for cumulative out-of-sample prediction when the regional degrees of freedom (described in the Material and Methods) are 29, 21, and 13 for height, BMI, and WHRadjBMI, respectively. The prediction performance of SOJO starts dropping after the regional degrees of freedom increases to 21 and 13 for BMI and WHRadjBMI, but does not drop for height even when the regional degrees of freedom increase to 25. This indicates that the allelic heterogeneity of height is the highest and that it is followed by BMI and WHRadjBMI for their established loci. This ranking is the same as the ranking of the estimated heritability^23^, ^24^ and the ranking of the number of loci detected in GIANT papers for the three traits.^6^, ^7^, ^8^ The same analysis was also performed with LD correlations estimated from the 503 European-ancestry samples in the 1000 Genomes Project phase 3 data, and the results are consistent (Figure 3).

When the variable selection thresholds were chosen as those maximizing locus-specific out-of-sample prediction $R^{2}$, SOJO again achieved larger out-of-sample prediction $R^{2}$ than top-SNPs-only prediction and GCTA-COJO (Table 1). If we take height as an example, the maximum proportion of phenotypic variance captured by the 423 locus-specific polygenic scores is 22.7% for SOJO and 21.4% for optimized GCTA-COJO. Compared to 13.8% achieved by the use of top variants only, the amount achieved by SOJO represents an increase of 65% over the out-of-sample prediction $R^{2}$. The amount of phenotypic variance captured by polygenic scores is consistent with the maximum cumulative out-of-sample prediction $R^{2}$, which indicates that these polygenic scores are almost independent.

**Table 1:** Maximum Phenotypic Variance Explained by Optimized Polygenic Scores and the Maximum Cumulative Out-of-Sample Prediction $R^{2}$ for SOJO and GCTA-COJO in UKB

	**Cumulative Prediction**$R^{2}$**(%)**				$R^{2}$**Explained by Polygenic Scores (%)**			
**Trait**	**Top Variant**	**Standard COJO**	**Optimized COJO**	**SOJO**	**Top Variant**	**Standard COJO**	**Optimized COJO**	**SOJO**

Height	14.35	17.38	23.42	24.52	13.76	16.71	21.42	22.70
BMI	1.88	1.99	2.42	2.52	1.84	1.94	2.35	2.46
WHRadjBMI	1.62	1.78	2.18	2.32	1.58	1.76	2.07	2.28

The $R^{2}$ for cumulative out-of-sample prediction was computed from a summation of all regional prediction $R^{2}$. $R^{2}$ explained by polygenic scores were the amount of phenotypic variance that could be explained by all regional polygenic scores.

Top variant: only the top variant was selected. Standard COJO: variants selected by COJO with $5\times 10^{-8}$ as the threshold. Optimized COJO: variants selected by COJO with threshold maximizing regional prediction $R^{2}$. Coefficients of variants in each polygenic score were estimated by joint multiple regression in COJO. SOJO: variants selected by LASSO with tuning parameter maximizing regional prediction $R^{2}$. Coefficients of variants in each polygenic score were determined by the LASSO result at the tuning parameter.

### SOJO Reveals Allelic Heterogeneity of Height

There are a number of reasons SOJO might achieve better prediction than GCTA-COJO. First, SOJO detects more underlying causal variants, whereas GCTA-COJO missed these causal variants because of the lower sensitivity under LD. Second, both methods detect variants tagging the same set of causal variants at a locus, but SOJO detects more variants capturing the information in these causal variants. Third, SOJO produces better effect estimates for prediction by using shrinkage estimators. In terms of biology, the first case is the most interesting. Therefore, we did a subsequent analysis to test the existence of the first scenario, i.e., that SOJO detected signals from additional causal variants. We performed this analysis on the 423 height loci by using individual-level data in UKB.

**Figure 4:**
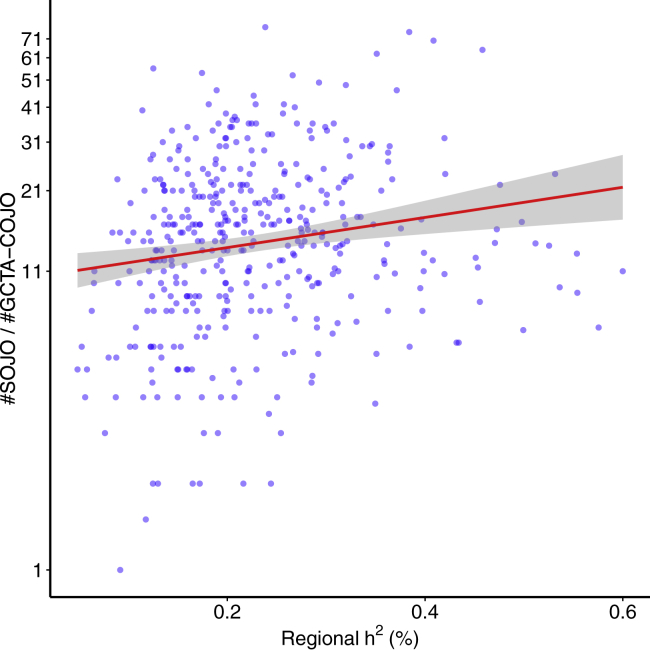
The Ratio of the Number of Variants Selected by SOJO to That Selected by GCTA-COJO in Terms of Height in UKB Tends to Increase as Regional $h^{2}$ Increases The plot is in logarithmic scale, and the y axis is labeled in the original scale. Regional $h^{2}$ is the multivariate regression $R^{2}$ when all variants at the locus are used. Each dot represents a locus. The red solid line represents the regression line in logarithmic scale. The gray shade denotes the 95% confidence interval for predicted mean values.

In principle, if GCTA-COJO does not miss any causal variant, and if we perform LASSO for each locus by using cross-validation and set a fixed p value threshold, e.g., $5\times 10^{-8}$ for GCTA-COJO, the ratio of the number of selected variants obtained with LASSO to that obtained with GCTA-COJO should not be affected by the allelic heterogeneity of the loci. However, higher allelic heterogeneity would lead to a larger possibility of the existence of correlated causal variants. If LASSO has a greater ability to detect causal variants in LD than GCTA-COJO, the ratio of the number of selected variants, i.e., the number of variants selected by LASSO/the number of variants selected by GCTA-COJO per locus, should increase with allelic heterogeneity. Because the allelic heterogeneity of each genetic locus is unknown and the genetic effects across the genome are very small, we used regional heritability $\left(h^{2}\right)$ as a proxy of allelic heterogeneity. The reasonability of $h^{2}$ as a proxy of allelic heterogeneity can be validated statistically: regional $h^{2}$ is significantly correlated with both the number of variants selected by LASSO and the number selected by GCTA-COJO (when $5\times 10^{-5}$ is taken as threshold for GCTA-COJO). The correlation coefficients are 0.61 (p = 1.8 × 10^−43^) and 0.62 (p = 2.3 × 10^−44^). In terms of choosing a proper threshold for GCTA-COJO, a strict threshold will make LASSO results dominate the ratio, whereas a loose threshold will generate lots of noise (Figure S3). In our analysis, because there are 165 variants in each region on average, we chose $5\times 10^{-5}$ as the cut-off, which is loose but still stricter than a 5% significance threshold after Bonferroni correction $\left(0.05/165=3\times 10^{-4}\right)$. The logarithm of the ratio increases significantly with regional $h^{2}$ (Figure 4) (slope of the regression = 1.21, p = 4.7 × 10^−4^), i.e., for a locus that has 0.1% more regional $h^{2}$ than another, the ratio is 1.13 times as large. This significantly positive slope suggests that GCTA-COJO missed some causal variants but that SOJO detected them or additional variants tagging them, and the amount is likely to be bigger when the allelic heterogeneity of the locus is larger. Therefore, the number of variants selected by SOJO is thus a better indicator of the locus-specific allelic heterogeneity. The same analysis was also done for BMI and WHRadjBMI. However, because the numbers of established loci are limited for these two traits, randomness dominated the correlation signal between the number of additional SOJO variants and regional $h^{2}$ (Figures S4 and S5).

## Discussion

We introduced a selection operator, SOJO, that analyzes multi-variant summary association statistics and is based on approximate LASSO shrinkage estimators. SOJO is more powerful than conditional and joint analysis in GCTA in terms of both discovery and prediction. SOJO is computationally fast because it is based on GWAS meta-analysis summary statistics and LD structure estimated from a reference cohort (Table S2). The small effects of genetic variants on complex traits imply that using estimates based on large-scale GWAS meta-analysis can substantially improve the precision of SOJO estimates, which provides a powerful tool for improving variant detection and better estimating genetic effects, especially in loci with LD. In future studies, SOJO might be useful for detecting more associated variants per locus in large-scale GWAS meta-analysis, providing better prediction based on detected loci, and suggesting allelic heterogeneity of complex traits.

As in GCTA-COJO, the reference sample is assumed to be from the same population where the meta-analysis sample is from. Therefore, a subcohort involved in the meta-analysis is usually valid as a reference sample. However, an outside sample can also be a reference sample if it well represents the population of interest. The sample size of the reference sample should be large enough so that the LD correlations can be estimated accurately. According to a simulation result by Yang et al.,^4^ a reference sample with more than 5,000 individuals is sufficient for achieving good accuracy. However, we were careful when using the estimated LD structure to get LASSO results: even though it is possible to implement SOJO on all the variants across the genome, we only applied it regionally. One main reason was that LASSO is more sensitive to the correlations between variants than COJO is, which is also why LASSO achieves better sensitivity and specificity when LD exists. Because of this characteristic, although the LASSO model can stably add top variants at the beginning of the selection procedure, as more and more variants are included, accumulated errors start disturbing the estimates. If SOJO is applied regionally, a relatively small publicly accessible sample such as 1000 Genomes can still be valid as a reference sample.

In many regions, SOJO top variants were also selected by GCTA-COJO (Table S1). This is expected because both perform variable selection based on partial correlations. However, it is hard for GCTA-COJO to include more informative variants in its model, especially when the p value threshold is less stringent. The first problem is specificity. As we lower the threshold (and increase the p value threshold), COJO includes more noise than signals. Overfitting is the consequent second problem. Without shrinkage, noise degrades the prediction. But for LASSO, because of shrinkage estimation, both problems are less serious, so LASSO can utilize more information in a genomic locus to obtain better prediction performance as a reward of avoiding overfitting. When the underlying causal variant is multi-allelic (such as with a short-tandem-repeat variation) instead of biallelic, SOJO tends to select multiple tagging SNPs for the causal variant. By doing this, it can better tag the latent multi-allelic causal variant and improve the prediction performance (Figure S6).

Evidence shows that jointly analyzing multiple correlated traits can improve both discovery power^25^, ^26^ and prediction performance.^27^ The possibility of extending LASSO to the multivariate context has been discussed in previous literature.^28^, ^29^ It is noteworthy that these multivariate LASSO methods, when applied on GWAS data, asymptotically only depend on (1) the LD correlation matrix, (2) the covariance structure between the phenotypes and genotypes, and (3) the covariance structure among the traits. These can all be estimated from summary association statistics and a reference cohort. Therefore, it is possible to extend SOJO to a multivariate framework in further studies.

Here, we use UKB, an independent individual-level data sample, as the validation sample to determine a proper amount of regularization or a reasonable number of variants selected for each locus. If there is no available independent sample, we suggest the use of the reference sample as the validation sample. Although the reference sample is used for estimating LD structure and was included in the GWAS meta-analysis where the summary statistics were from, it can still function as a validation sample because it usually contributes only a little to the estimation of genetic effects. Our method can also be used directly with individual-level data where the reference sample is the whole cohort. In this case, SOJO is equivalent to standard LASSO that is based on individual-level data. If the summary statistics and LD structure have been computed and stored beforehand, SOJO is computationally faster than standard LASSO. Another benefit of SOJO is its ability to handle variants with unequal sample size. This means that when individuals or individual cohorts have missing genotype data, SOJO is able to take this into account to estimate correlations between the variants and the trait instead of removing valuable individuals because of missing data.

According to our empirical results, human height not only is a highly polygenic trait but also has high allelic heterogeneity. This interesting coincidence might be due to assortative mating; i.e., individuals prefer partners with similar phenotypes.^30^ Assortative mating will increase the proportion of homozygous progeny and prevent the alleles of trait-associated variants from drifting away. A recent study inferred a correlation between trait-associated loci for height (0.200, 0.004 SE), BMI (0.143, 0.007 SE), and waist-to-hip ratio (0.101, 0.041 SE) in partners.^31^ This ranking is consistent with the ranking of allelic heterogeneity levels in our results for the three traits.

The tuning parameter in LASSO is usually chosen by cross-validation, which is impossible for SOJO because the individual-level data of the GWAS meta-analysis are absent. Variant selection based on one validation sample might be less stable than standard cross-validation. Hence, SOJO could be improved by using the validation sample more thoroughly via splitting or bootstrapping. If one would like to avoid using the validation sample, but use only GWAS summary statistics and the reference sample, some additional methods are worthy of investigation. Although the individual-level data of the GWAS meta-analysis is absent, making it impossible to bootstrap the individuals, one could perform a parametric Monte Carlo simulation on the estimated genetic effects, given that their point estimates and standard errors are available from summary association statistics and their correlations can be estimated from the reference sample. With Monte Carlo simulation results, we can improve the variant detection and phenotypic prediction of SOJO further by implementing bootstrap-based methods such as stability selection^32^ or Bolasso.^33^ These methods might also be helpful for determining the LASSO tuning parameter when the validation sample is unavailable.

## Web Resources

SOJO, https://github.com/zhenin/sojo
GIANT summary association statistics, http://portals.broadinstitute.org/collaboration/giant/index.php/GIANT_consortium_data_files
Genotypes of 1000 Genomes European-ancestry samples, ftp://ftp.1000genomes.ebi.ac.uk/vol1/ftp/data_collections/1000_genomes_project/data

## Supplemental Data

Document S1. Figures S1–S6 and Table S2

Table S1. Summary of Jointly Associated SNPs Suggested by SOJO

Document S2. Article plus Supplemental Data
