# Supplementary material for: A Selection Operator for Summary Association Statistics Reveals Allelic Heterogeneity of Complex Traits
Source: Am J Hum Genet. 2017 Dec 5;101(6):903–12. doi: 10.1016/j.ajhg.2017.09.027 (PMC5812891; doi:10.1016/j.ajhg.2017.09.027)
Supplement: Document S1. Figures S1–S6 and Table S2 [file mmc1.pdf]

The American Journal of Human Genetics, Volume *101*

## Supplemental Data

# A Selection Operator for Summary Association Statistics Reveals Allelic Heterogeneity of Complex Traits

**Zheng Ning, Youngjo Lee, Peter K. Joshi, James F. Wilson, Yudi Pawitan, and Xia Shen**

Figure S1: Examples of the degrees freedom for SOJO and GCTA-COJO

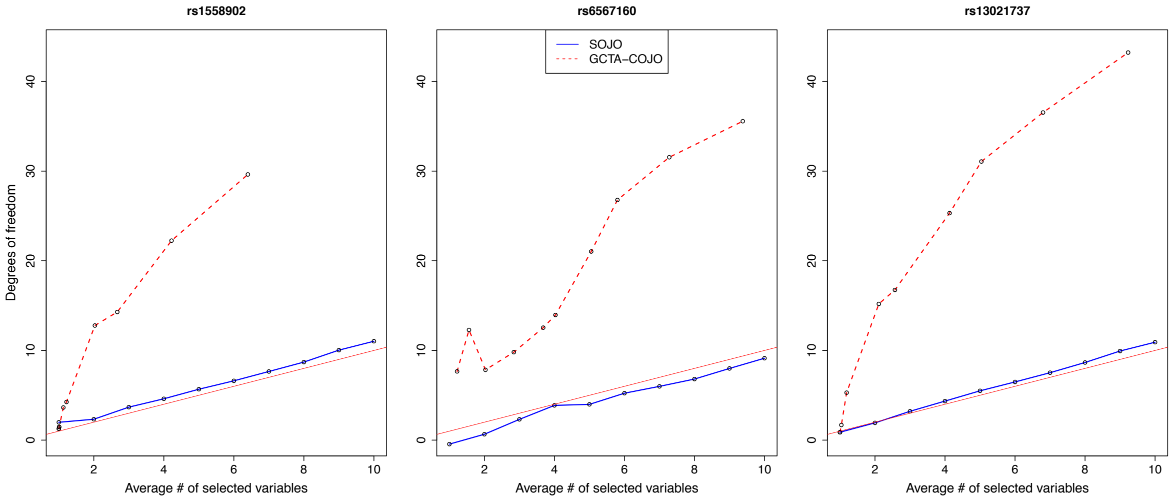


The GDF estimation performed on the first three BMI-associated loci in GIANT. Using Algorithm 1 in Ref 23, the GDF for SOJO and GCTA-COJO were estimated separately by 100 simulations at different model-selection threshold. These plots show the estimated degrees of freedom and average number of selected variables by SOJO (blue solid) and GCTA-COJO (red dashed). The red solid line is the diagonal $x = y$ line. The GDF of SOJO lines up with the average number of selected variables, while the GDF of GCTA-COJO is often larger.

Figure S2: Prediction performances of situations in Figure 1 using UKB height data

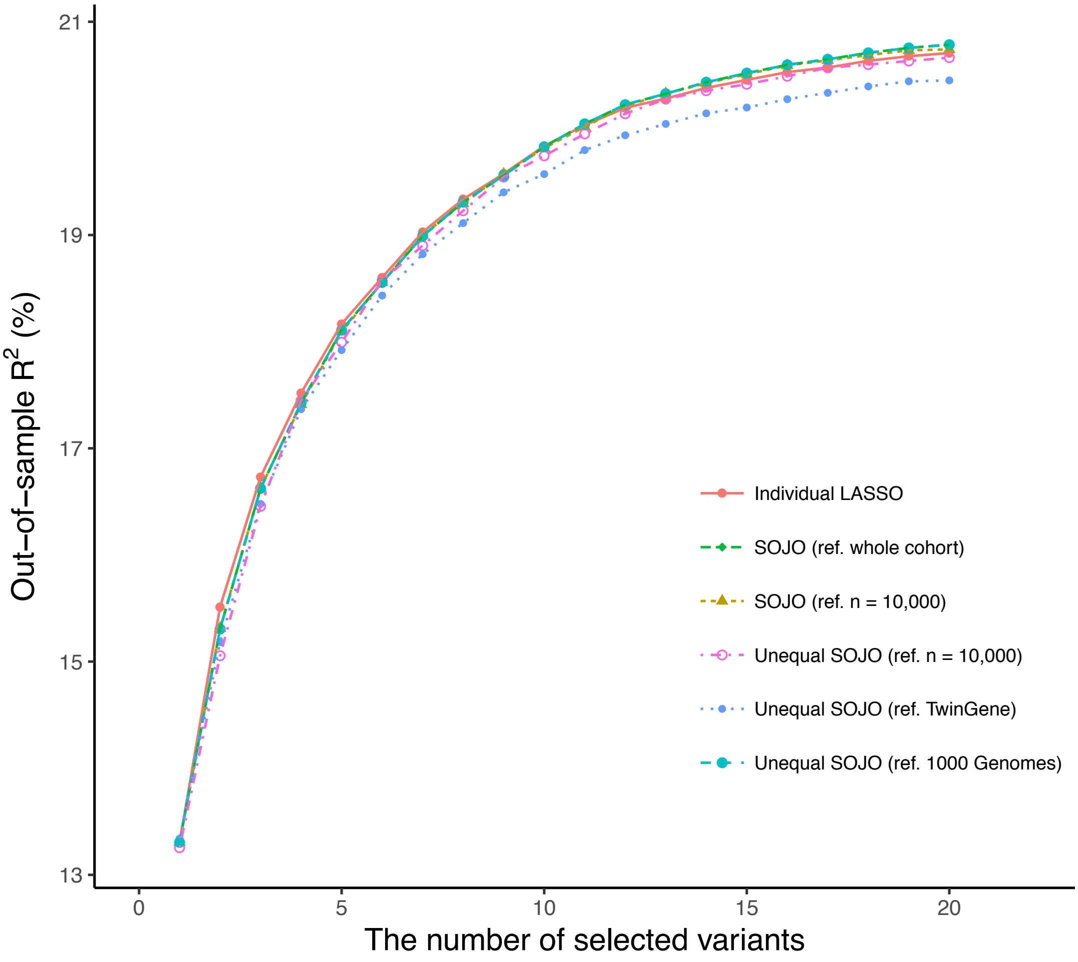


Half of UKB samples (n = 60,043) is used as training set and the other half is as test set. Cumulative prediction $R^2$ was computed in the same way as Figure 3 (See Results part). The situations ordered in legend correspond to case (A) to (F) in Figure 1.

Figure S3: The ratio of the number of selected variants using SOJO to GCTA-COJO on height in UKB at different p-value thresholds for COJO

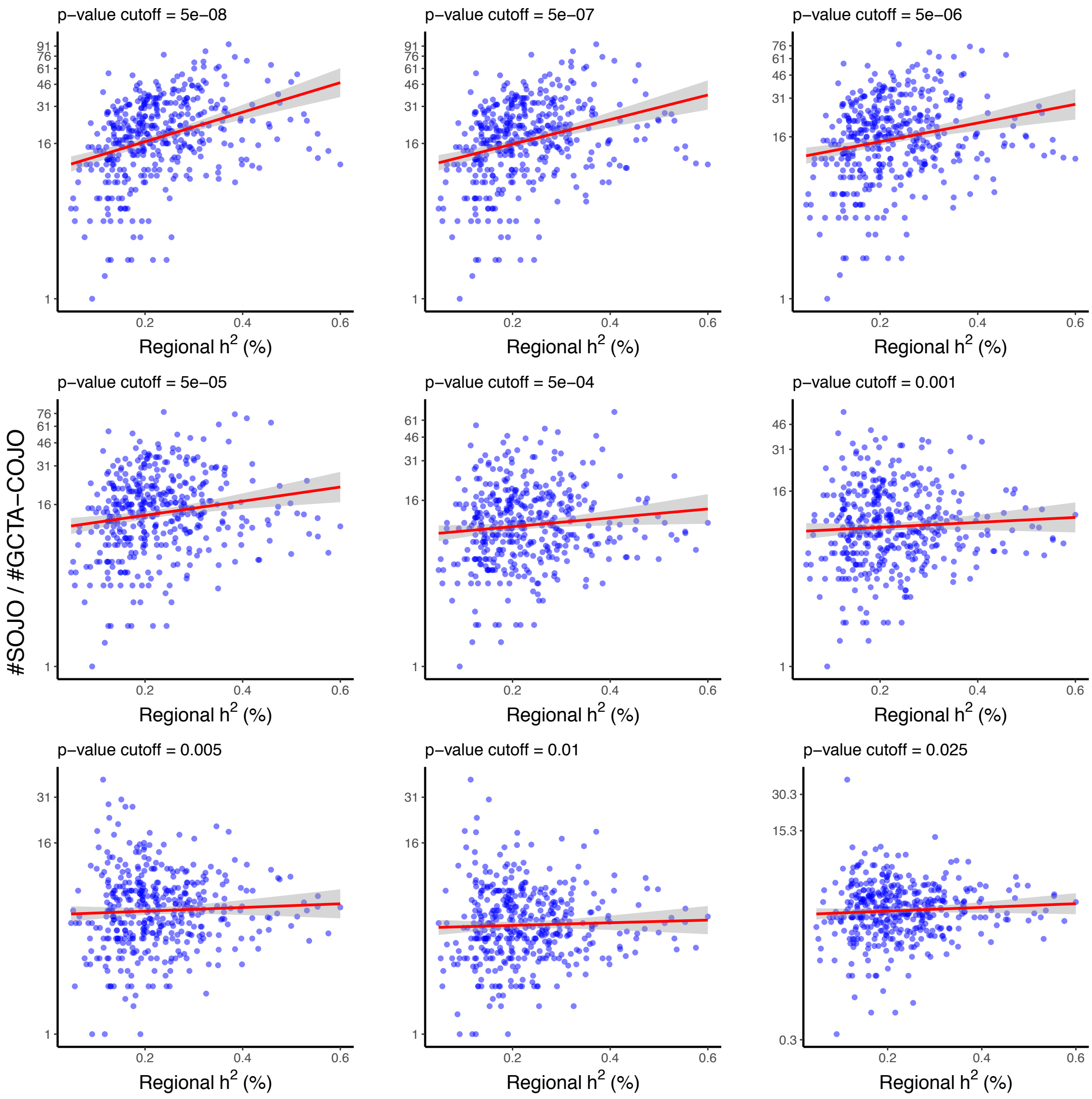


The plot is in logarithmic scale with y axis label in original scale. Regional $h^2$ is the multivariate regression $R^2$ using all variants at the locus. Each dot represents a locus. The red solid line gives the regression line in logarithmic scale. The grey shade denotes the 95% confidence interval for predicted mean values.

Figure S4: The ratio of the number of selected variants using SOJO to GCTA-COJO on WHRadjBMI in UKB at different p-value thresholds for COJO

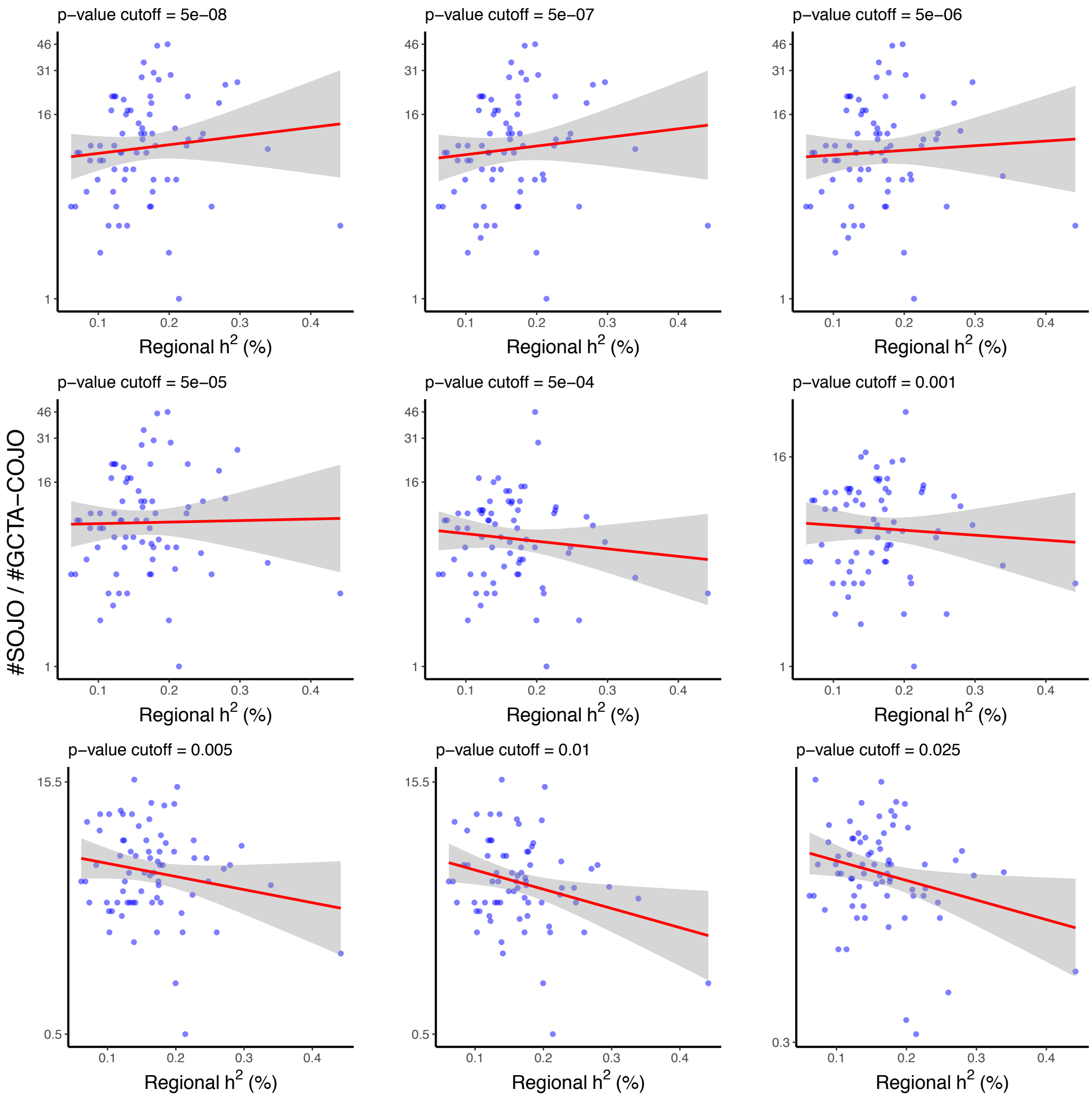


The plot is in logarithmic scale with y axis label in original scale. Regional $h^2$ is the multivariate regression $R^2$ using all variants at the locus. Each dot represents a locus. The red solid line gives the regression line in logarithmic scale. The grey shade denotes the 95% confidence interval for predicted mean values.

Figure S5: The ratio of the number of selected variants using SOJO to GCTA-COJO on WHRadjBMI in UKB at different p-value thresholds for COJO

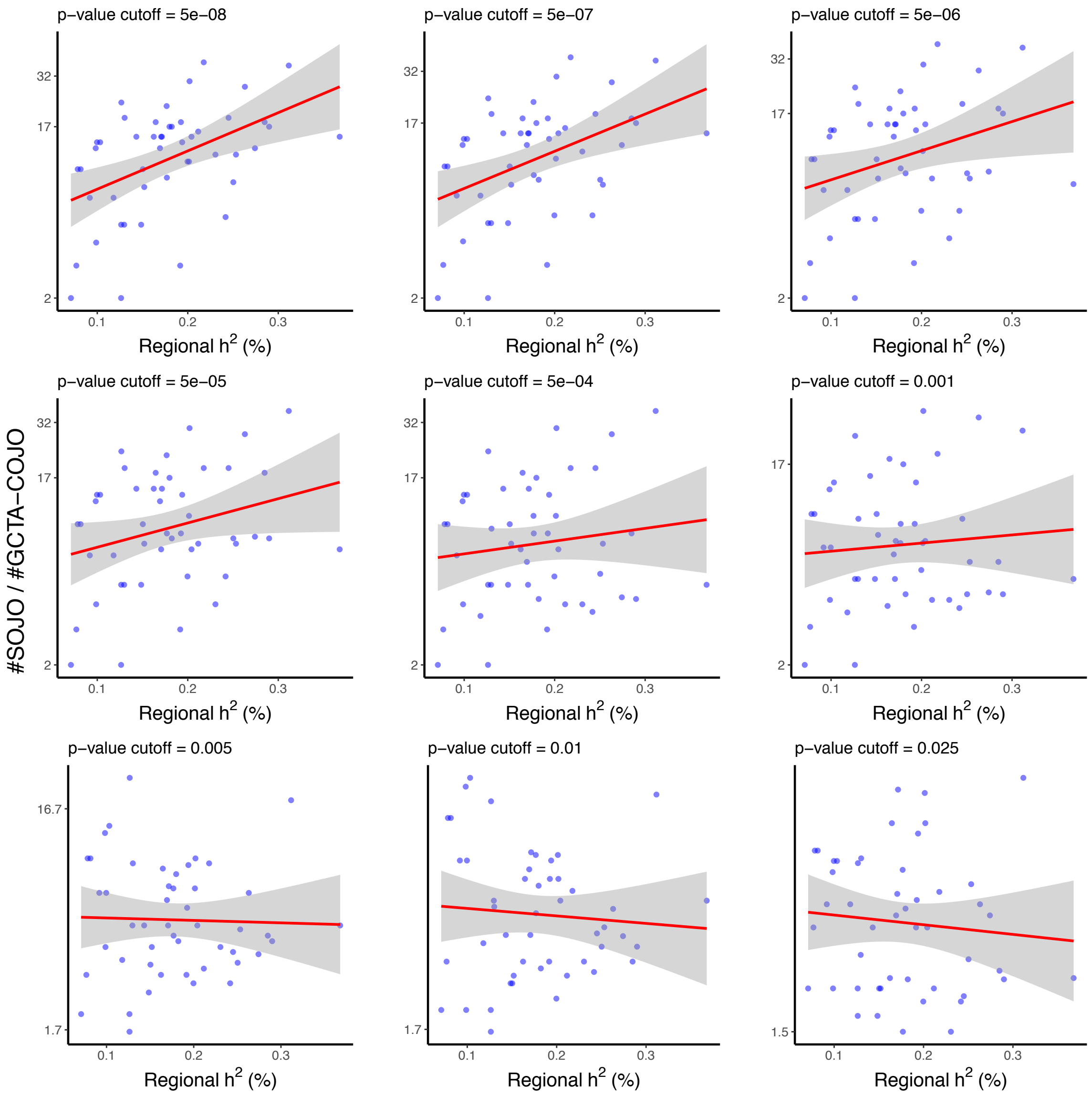


The plot is in logarithmic scale with y axis label in original scale. Regional $h^2$ is the multivariate regression $R^2$ using all variants at the locus. Each dot represents a locus. The red solid line gives the regression line in logarithmic scale. The grey shade denotes the 95% confidence interval for predicted mean values.

Figure S6: The prediction performance of SOJO when the latent causal variant is multiallelic

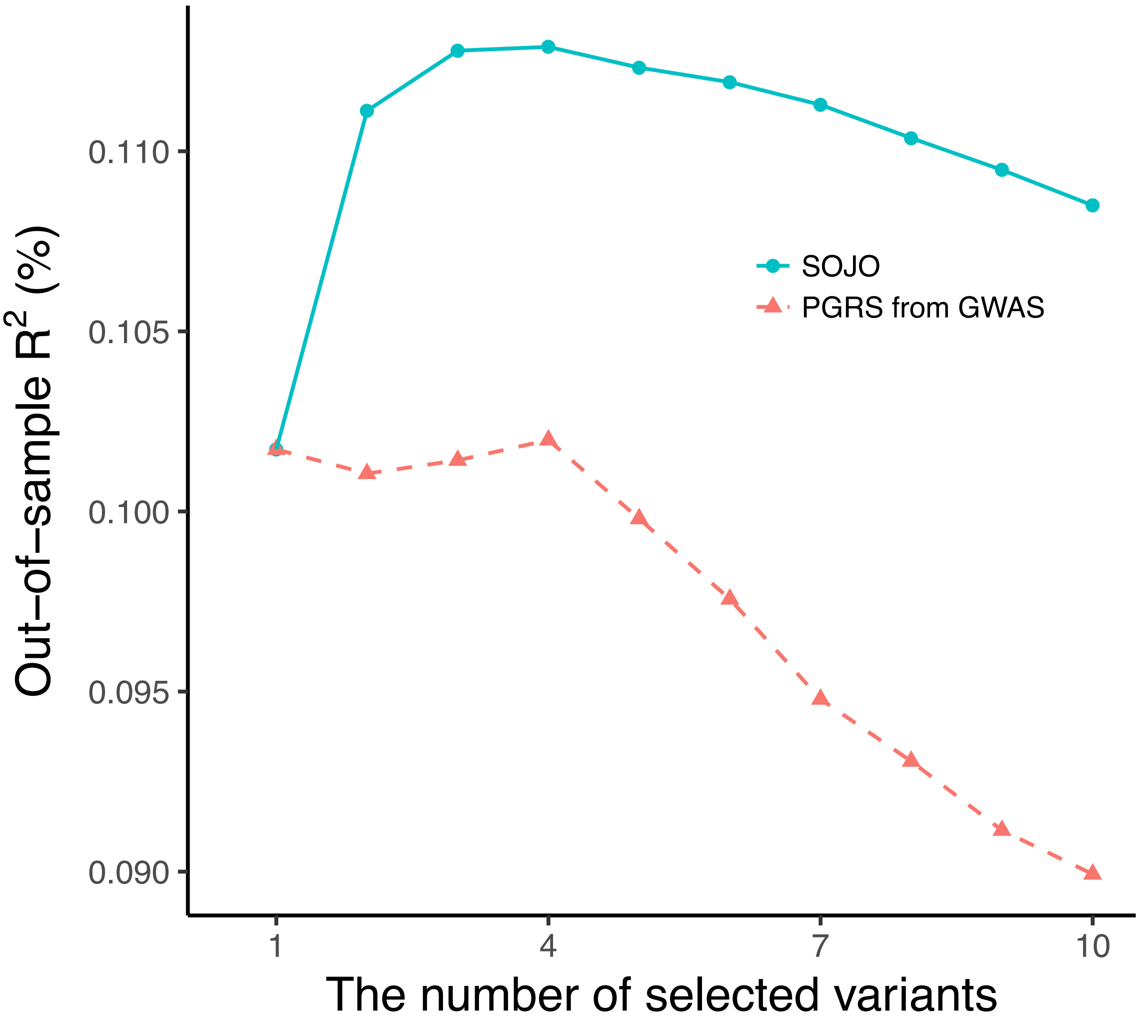


The solid blue line represents the prediction performance of the polygenic scores based on SOJO. The dashed red line is from polygenic scores based on GWAS top variants. Datasets were simulated for 100,000 individuals with 20 variants, where the 10th variant is the causal CNV whose value is from 0 to 5. All variants follow $B(2, 0.5)$ except the 10th variant follows $B(5, 0.8)$. The effects of the causal variant are non-additive: For CNV value from 0 to 5, the effects are (0,1,2,2,6,8) separately. $\mathrm{cor}(X_i, X_j) = 0.7^{|i-j|}$ except $\mathrm{cor}(X_{10}, X_{11}) = 0.5$

so that the particular symmetric LD structure around the causal variant is broken. The trait $y = f(X_{10}) + e$, where $f$ is the effect correspondence function described above and $e \sim N(0, 50^2)$. The causal variant is hidden after generating trait. 1,000 datasets were generated. In each dataset, there are a training set and a test set. In both sets, for $k$ from 1 to 10, SOJO selected $k$ variants and estimated their effect size, then built a polygenic score. We also run GWAS and used $k$ top variants and their estimated coefficients to get another polygenic score. Then the out-of-sample $R^2$ of these polygenic scores were computed in test set. The points represent the median result of 1,000 datasets.

Table S1: Summary of jointly associated SNPs suggested by SOJO. Using TwinGene as reference sample, GIANT summary statistics as training data, and UKB as validation sample, we suggested 8,470, 1,026 and 522 jointly associated variants by implementing SOJO on 423, 77 and 49 established loci for height, BMI and WHRadjBMI respectively. For both SOJO and COJO, we reported the variants and their effect sizes at the thresholds maximizing the out-of-sample $R^2$. Top SNP, the most significant SNP at the locus in GIANT; Nearest Gene, the gene that is closest to the top SNP; Chr., chromosome; Interval, the 1-Mb window centred at the top SNP; Regional h2, variance explained based on all SNPs in the interval; Out of sample R2 top, prediction $R^2$ based on the top SNP; Out of sample R2 GCTA-COJO, prediction $R^2$ based on the polygenic score built with COJO-selected variants and their effect sizes; Out of sample R2 SOJO, prediction $R^2$ based on the polygenic score built with SOJO-selected variants and their penalized effect sizes; COJO threshold, p-value thresholds where COJO polygenic score maximizes the out-of-sample $R^2$; Variants (COJO), variants selected by GCTA-COJO given the threshold; r, correlation between the SNP and top SNP; Joint beta(se), joint effect and standard error in GCTA-COJO result; Variants (SOJO), variants selected by SOJO. A variant starts with an asterisk if it is also selected when LD matrix is estimated from 1000 Genome; LASSO beta, penalized effect in SOJO result.

Table S2: Computing speed of SOJO and GCTA-COJO

| Number of variants | GCTA-COJO | | | SOJO | |
|---|---|---|---|---|---|
| | P-value threshold | Number of selected markers | Time ± SE (s) | Number of selected markers | Time ± SE (s) |
| 200 | $5 \times 10^{-8}$ | 2 | $0.110 \pm 0.001$ | 10 | $0.010 \pm 0.001$ |
| | $5 \times 10^{-4}$ | 19 | $0.214 \pm 0.002$ | 50 | $0.057 \pm 0.003$ |
| | $5 \times 10^{-2}$ | 66 | $0.621 \pm 0.003$ | 100 | $0.234 \pm 0.006$ |
| 567 | $5 \times 10^{-8}$ | 1 | $0.222 \pm 0.006$ | 10 | $0.052 \pm 0.007$ |
| | $5 \times 10^{-4}$ | 9 | $0.252 \pm 0.002$ | 50 | $0.101 \pm 0.004$ |
| | $5 \times 10^{-2}$ | 60 | $1.861 \pm 0.009$ | 100 | $0.367 \pm 0.009$ |

Two regions with 200 and 567 variants separately were selected to test computing speed. For each method and each threshold, 100 repetitions were performed to get the mean computing speed and its standard error. Number of variants, the number of variants in the region; P-value threshold, the p-value threshold used by GCTA-COJO; Number of selected markers, the number of variants selected by the method (the number is given by result for GCTA-COJO, and preset for SOJO); Time ± SE, the mean time consumption for one computation of the method, and the standard error of the mean. The unit is second.
